# Associations between the components of metabolic syndrome and the polymorphisms in the peroxisome proliferator-activated receptor gamma (*PPAR-γ*), the fat mass and obesity-associated (*FTO*), and the melanocortin-4 receptor (*MC4R*) genes

**DOI:** 10.18632/aging.101360

**Published:** 2018-01-09

**Authors:** Małgorzata Szkup, Aleksander Jerzy Owczarek, Daria Schneider-Matyka, Jacek Brodowski, Beata Łój, Elżbieta Grochans

**Affiliations:** 1 Department of Nursing, Pomeranian Medical University in Szczecin, 71-210 Szczecin, Poland; 2 Department of Statistics, Department of Instrumental Analysis, School of Pharmacy with the Division of Laboratory Medicine in Sosnowiec, Medical University of Silesia, Katowice, 41-200 Sosnowiec, Poland; 3 Primary Care Department, Pomeranian Medical University in Szczecin, 71-210 Szczecin, Poland; 4 Klinik für Gynäkologie und Geburtshilfe, Sana HANSE-Klinikum Wismar GmbH, 23966 Wismar, Germany

**Keywords:** PPAR gamma, receptor, melanocortin, type 4, FTO protein, human, metabolic syndrome X

## Abstract

Introduction: Metabolic syndrome (MetS) is regarded as a set of abnormalities, increasing the risk of serious functioning disorders. It can develop as a result of genetic predisposition.

Aim: The aim of this study was to establish associations between MetS-related abnormalities and the *PPAR-γ* rs1801282, *FTO* rs9939609, and *MC4R* rs17782313 polymorphisms.

Material and methods: The study involved 425 women aged 45-60 years. The participants were surveyed and subjected to anthropometric, biochemical and genetic analysis.

Results:In the recessive inheritance model for the *FTO* polymorphism, a statistically significant relationship was demonstrated between the A/A genotype and glycemia. The results obtained in the codominant and overdominant models for the *PPAR-y* polymorphism showed a tendency to statistical significance (the C/G genotype inclined to hypertriglyceridemia), and were statistically significant in the codominant, dominant, and recessive models (the C/C genotype predisposed to increased blood pressure).

Conclusions: 1. MetS-related abnormalities can be genetically determined, however only some of these relationships can be demonstrated due to the categorical division of symptoms according to the IDF criteria from 2009. 2. The A/A genotype of the *FTO* rs9939609 polymorphism increases the risk of hyperglycemia, and the C/C genotype of the *PPAR-γ* rs1801282 variant entails elevated blood pressure in 45-60-year-old women.

## Introduction

Metabolic syndrome (MetS) is a complex of interrelated abnormalities, increasing the risk of diabetes and cardiovascular disease. Its pathogenesis, pondered mainly in the context of behavioral and environmental influences, has not yet been fully explicated.

There is some divergence in medical circles as for terminology, definition, and diagnostic criteria for identification of MetS patients. Some clinicians take into account the possibility that MetS should not be treated as a separate syndrome but rather as a mixture of unrelated phenotypes. However, as a set of symptoms that occur together definitely more often than separately, MetS seems to meet the requirements of the definition. Not including such factors as age, the low-density lipoprotein (LDL) cholesterol level, and smoking ― which unquestionably contribute to the risk of cardiovascular disease ― the current MetS definition cannot be regarded as the index of absolute risk [1]. Nevertheless, studies show that people who meet MetS criteria are at a five-fold higher risk of developing type 2 diabetes, a two-and-half-fold higher risk of death for acute coronary syndrome, a twice higher risk of stroke, and their total mortality rate is one-and-half-fold higher than in the general population [2,3].

Apart from environmental factors and lifestyle ― whose contribution to MetS is indisputable ― some responsibility can also be attributed to genetic determinants, though their role has not so far been fully elucidated. This assumption is supported by screening results, showing that MetS is inherited in 10–30% of cases [4]. The risk for MetS is mainly associated with point mutations (deletion, insertion, or substitution to a single nucleotide) [5]. It is believed that genetic predisposition to MetS can be related to polymorphic forms of genes, playing an important part in the expression of MetS components [6]. Hence our decision to analyze relationships between particular components of MetS and selected gene polymorphisms, namely peroxisome proliferator-activated receptor gamma (*PPAR-γ*), fat mass and obesity-associated (*FTO*), and melanocortin-4 receptor (*MC4R*).

As suggested by research outcomes, *PPAR-γ* rs1801282 is essential for lipid metabolism, adipogenesis, and maintenance of glucose homeostasis in serum. It also has effects on the level of insulin resistance, as well as on inflammation and cancer processes [7,8]. *PPAR-γ* may potentially play a role in the development of hypertension through its involvement in the regulation of vascular tension [9]. What is more, activation of *PPAR-y* by rosiglitazone increases glucose uptake in muscle cells and adipocytes, and consequently reduces the level of glycemia in plasma. This is an effect of higher expression and translocation of the glucose transporter 1 (GLUT1) and the glucose transporter 4 (GLUT4) [10]. Lower triglycerides (TG) and higher HDL levels can also enhance the effect of the *PPAR-y* agonists on the development of MetS, atherosclerosis and cardiovascular complications [11].

The *FTO* rs9939609 polymorphism, located in people in chromosome region 16q12.2, is responsible for encoding nucleic acid demethylase, which is crucial for energy balance control in the body [12]. Scientists believe that polymorphic *FTO* variants can influence the process of maintaining energy homeostasis, and the control of energy expenditure [13]. Previous genetic studies led to contradictory results. Some of them demonstrated that the *FTO* polymorphisms are related to body mass index (BMI) and obesity, and strongly contribute to MetS-related abnormalities [12,14,15]. Other reports suggest that such relationships do not exist [16,17]. It seems highly probable that the *FTO* is a race-specific gene. The study conducted by Lear et al. revealed that the A allele, regarded as the risk allele for the development of MetS-related abnormalities, was most common among Europeans (39%) and South Asians (31%), and present only in 17% of Aboriginals and 17% of Chinese [18].

*MC4R* rs17782313 is a G-protein-coupled receptor. It has effects on the central nervous system, regulation of dietary habits, control of the TG synthesis, energetic balance in the body, as well as accumulation of lipids and their mobilization in white adipose tissue (WAT) [19,20]. Located in chromosome region 18q22, polymorphic variants of the *MC4R* gene contribute to body weight disorders [21].

The aim of this study was to analyze association between MetS-related abnormalities and the *PPAR-γ* rs1801282, the *FTO* rs9939609, and the *MC4R* rs17782313 gene polymorphisms.

## RESULTS

Analysis of the distribution of MetS-related abnormalities, according to the modified IDF criteria from 2009, demonstrated that only 27.06% of the study sample (425 women aged 45-60 years) had normal waist size. Fasting glycemia within normal ranges was observed in 75.53%, normal TG metabolism in 78.12%, and normal HDL levels were found in 74.06%. 46.82% of the participants had systolic blood pressure (sRR) < 130 mmHg, and 70.12% had diastolic blood pressure (dRR) < 85mmHg.

We analyzed different models of inheritance (codominant, dominant, recessive, and overdominant) for the *PPAR-γ*, *FTO* and *MC4R* genotypes with regard to five MetS symptoms. No statistically significant relationships were noticed between particular inheritance models and waist size (Table 1).

**Table 1:** Odds ratios (OR) calculated assuming different models of inheritance of *PPARγ rs1801282*, *FTO rs9939609* and *MC4R* *rs17782313* SNPs with regard to waist size (**S_1_^-^** = <80cm; **S_1_^+^** = ≥80cm).

Genotype ***PPARy***	**S_1_^-^**	**S_1_^+^**	**OR**	**p**	Genotype ***FTO***	**S_1_^-^**	**S_1_^+^**	**OR**	**p**	Genotype ***MC4R***	**S_1_^-^**	**S_1_^+^**	**OR**	**p**
**Co-dominant**														
C/C	214 (68,6%)	79 (71,2%)	1,00	0,88	T/T-A/T	110 (35%)	34 (30,6%)	1,00	0,68	T/T	201 (64%)	73 (65,8%)	1,00	0,54
C/G	80 (25,6%)	26 (23,4%)	0,88 (0,53-1,47)		A/T	143 (45,5%)	55 (49,5%)	1,24 (0,76-2,04)		C/T	107 (34,1%)	34 (30,6%)	0,87 (0,55-1,40)	
G/G	18 (5,8%)	6 (5,4%)	0,90 (0,35-2,36)		A/A	61 (19,4%)	22 (19,8%)	1,17 (0,63-2,17)		C/C	6 (1,9%)	4 (3,6%)	1,84 (0,50-6,69)	
**Dominant**														
C/C	214 (68,6%)	79 (71,2%)	1,00	0,61	T/T	110 (35%)	34 (30,6%)	1,00	0,40	T/T	201 (64%)	73 (65,8%)	1,00	0,74
C/G-G/G	98 (31,4%)	32 (28,8%)	0,88 (0,55-1,42)		A/T-A/A	204 (65%)	77 (69,4%)	1,22 (0,77-1,94)		C/T-C/C	113 (36%)	38 (34,2%)	0,93 (0,59-1,46)	
**Recessive**														
C/C-C/G	294 (94,2%)	105 (94,6%)	1,00	0,89	T/T-A/T	253 (80,6%)	89 (80,2%)	1,00	0,93	T/T-C/T	308 (98,1%)	107 (96,4%)	1,00	0,33
G/G	18 (5,8%)	6 (5,4%)	0,93 (0,36-2,41)		A/A	61 (19,4%)	22 (19,8%)	1,03 (0,60-1,77)		C/C	6 (1,9%)	4 (3,6%)	1,92 (0,53-6,93)	
**Overdominant**														
C/C-G/G	232 (74,4%)	85 (76,6%)	1,00	0,64	T/T-A/A	171 (54,5%)	56 (50,5%)	1,00	0,47	T/T-C/C	207 (65,9%)	77 (69,4%)	1,00	0,51
C/G	80 (25,6%)	26 (23,4%)	0,89 (0,53-1,47)		A/T	143 (45,5%)	55 (49,5%)	1,17 (0,76-1,81)		C/T	107 (34,1%)	34 (30,6%)	0,85 (0,54-1,36)	

The best models for particular genes were as follows: for PPAR-y - the dominant model; for FTO – the dominant model; for MC4R – the recessive model

Similar analysis of serum fasting glucose level and related pharmacotherapy revealed a statistically significant relationship between this symptom and the recessive inheritance model for the *FTO* polymorphism. In this model, the A/A genotype carriers were at higher risk of fasting hyperglycemia than those with the T/T-A/T genotypes (Table 2).

**Table 2:** Odds ratios (OR) calculated assuming different models of inheritance of *PPARγ* *rs1801282*, *FTO rs9939609* and *MC4R* *rs17782313* SNPs with regard to fasting glycemia (S_2_^-^ = <100 mg/dl; S_2_^+^ **=** ≥ 100 mg/dl or related pharmacotherapy).

Genotype ***PPARy***	**S_2_^-^**	**S_2_^+^**	**OR**	**p**	Genotype ***FTO***	**S_2_^-^**	**S_2_^+^**	**OR**	**p**	Genotype ***MC4R***	**S_2_^-^**	**S_2_^+^**	**OR**	**p**
**Co-dominant**														
C/C	203 (69,8%)	90 (68,2%)	1,00	0,65	T/T-A/T	106 (36,2%)	38 (28,8%)	1,00	0,075	T/T	193 (65,9%)	81 (61,4%)	1,00	0,65
C/G	70 (24,1%)	36 (27,3%)	1,16 (0,72-1,86)		A/T	138 (47,1%)	60 (45,5%)	1,21 (0,75-1,96)		C/T	93 (31,7%)	48 (36,4%)	1,23 (0,80-1,90)	
G/G	18 (6,2%)	6 (4,5%)	0,75 (0,29-1,96)		A/A	49 (16,7%)	34 (25,8%)	1,94 (1,09-3,43)		C/C	7 (2,4%)	3 (2,3%)	1,02 (0,26-4,05)	
**Dominant**														
C/C	203 (69,8%)	90 (68,2%)	1,00	0,74	T/T	106 (36,2%)	38 (28,8%)	1,00	0,13	T/T	193 (65,9%)	81 (61,4%)	1,00	0,37
C/G-G/G	88 (30,2%)	42 (31,8%)	1,08 (0,69-1,68)		A/T-A/A	187 (63,8%)	94 (71,2%)	1,40 (0,90-2,19)		C/T-C/C	100 (34,1%)	51 (38,6%)	1,22 (0,79-1,86)	
**Recessive**														
C/C-C/G	273 (93,8%)	126 (95,5%)	1,00	0,49	T/T-A/T	244 (83,3%)	98 (74,2%)	1,00	**<0,05**	T/T-C/T	286 (97,6%)	129 (97,7%)	1,00	0,94
G/G	18 (6,2%)	6 (4,5%)	0,72 (0,28-1,86)		A/A	49 (16,7%)	34 (25,8%)	1,73 (1,05-2,84)		C/C	7 (2,4%)	3 (2,3%)	0,95 (0,24-3,73)	
**Overdominant**														
C/C-G/G	221 (76%)	96 (72,7%)	1,00	0,48	T/T-A/A	155 (52,9%)	72 (54,5%)	1,00	0,75	T/T-C/C	200 (68,3%)	84 (63,6%)	1,00	0,35
C/G	70 (24,1%)	36 (27,3%)	1,18 (0,74-1,89)		A/T	138 (47,1%)	60 (45,5%)	0,94 (0,62-1,41)		C/T	93 (31,7%)	48 (36,4%)	1,23 (0,80-1,89)	

The best models for particular genes were as follows: for PPAR-y - the overdominant model; for FTO – the recessive model; for MC4R – the overdominant model

Elevated serum TG levels were not significantly associated with particular inheritance models. There were, however, relationships showing a tendency to statistical significance in the codominant model for the *PPAR-γ* polymorphism, in which the C/G genotype carriers were more likely to develop hypertriglyceridemia than those with the C/C and G/G genotypes. We also noticed that in the overdominant model for the same gene polymorphism, the C/G genotype predisposed to TG metabolism disorders more than the C/C-G/G genotypes (Table 3).

**Table 3:** Odds ratios (OR) calculated assuming different models of inheritance of *PPARγ* *rs1801282*, *FTO* *rs9939609* and *MC4R rs17782313* SNPs with regard to the serum TG level (S_3_^-^ = <150 mg/dl; S_3_^+^ **=** ≥ 150 mg/dl or related pharmacotherapy).

Genotype ***PPARy***	**S_3_^-^**	**S_3_^+^**	**OR**	**p**	Genotype ***FTO***	**S_3_^-^**	**S_3_^+^**	**OR**	**p**	Genotype ***MC4R***	**S_3_^-^**	**S_3_^+^**	**OR**	**p**
**Co-dominant**														
C/C	221 (70,6%)	72 (65,5%)	1,00	0,06	T/T-A/T	113 (35,9%)	31 (28,2%)	1,00	0,33	T/T	206 (65,4%)	68 (61,8%)	1,00	0,67
C/G	71 (22,7%)	35 (31,8%)	1,51 (0,93-2,46)		A/T	143 (45,4%)	55 (50,0%)	1,40 (0,85-2,32)		C/T	101 (32,1%)	40 (36,4%)	1,20 (0,76-1,90)	
G/G	21 (6,7%)	3 (2,7%)	0,44 (0,13-1,51)		A/A	59 (18,7%)	24 (21,8%)	1,48 (0,80-2,75)		C/C	8 (2,5%)	2 (1,8%)	0,76 (0,16-3,65)	
**Dominant**														
C/C	221 (70,6%)	72 (65,5%)	1,00	0,32	T/T	113 (35,9%)	31 (28,2%)	1,00	0,14	T/T	206 (65,4%)	68 (61,8%)	1,00	0,50
C/G-G/G	92 (29,4%)	38 (34,5%)	1,27 (0,80-2,01)		A/T-A/A	202 (64,1%)	79 (71,8%)	1,43 (0,89-2,29)		C/T-C/C	109 (34,6%)	42 (38,2%)	1,17 (0,74-1,83)	
**Recessive**														
C/C-C/G	292 (93,3%)	107 (97,3%)	1,00	0,09	T/T-A/T	256 (81,3%)	86 (78,2%)	1,00	0,49	T/T-C/T	307 (97,5%)	108 (98,2%)	1,00	0,66
G/G	21 (6,7%)	3 (2,7%)	0,39 (0,11-1,33)		A/A	59 (18,7%)	24 (21,8%)	1,21 (0,71-2,06)		C/C	8 (2,5%)	2 (1,8%)	0,71 (0,15-3,40)	
**Overdominant**														
C/C-G/G	242 (77,3%)	75 (68,2%)	1,00	0,06	T/T-A/A	172 (54,6%)	55 (50,0%)	1,00	0,41	T/T-C/C	214 (67,9%)	70 (63,6%)	1,00	0,41
C/G	71 (22,7%)	35 (31,8%)	1,59 (0,98-2,57)		A/T	143 (45,4%)	55 (50,0%)	1,20 (0,78-1,86)		C/T	101 (32,1%)	40 (36,4%)	1,21 (0,77-1,91)	

The best models for particular genes were as follows: for PPAR-y - the overdominant model; for FTO – the dominant model; for MC4R – the overdominant model

The inheritance models had no statistically significant impact on the serum HDL level. The relationship that was closest to the significance limit was that between the overdominant model for the *FTO* polymorphism and the HDL level ― the A/T genotype was associated with having HDL on a sufficiently high level. By contrast, the T/T-A/A genotypes were more often accompanied by the HDL level <50 mg/dl (Table 4).

**Table 4:** Odds ratios (OR) calculated assuming different models of inheritance of *PPARγ* *rs1801282*, *FTO* *rs9939609* and *MC4R rs17782313* SNPs with regard to the serum HDL level (S_4_^-^ = >50 mg/dl; S_4_^+^ **=** ≤ 50 mg/dl or related pharmacotherapy).

Genotype ***PPARy***	**S_4_^-^**	**S_4_^+^**	**OR**	**p**	Genotype ***FTO***	**S_4_^-^**	**S_4_^+^**	**OR**	**p**	Genotype ***MC4R***	**S_4_^-^**	**S_4_^+^**	**OR**	**p**
**Co-dominant**														
C/C	67 (65,7%)	226 (70,4%)	1,00	0,65	T/T-A/T	29 (28,2%)	115 (35,7%)	1,00	0,18	T/T	66 (64,1%)	208 (64,6%)	1,00	0,94
C/G	28 (27,4%)	78 (24,3%)	0,83 (0,50-1,38)		A/T	56 (54,4%)	142 (44,1%)	0,64 (0,38-1,07)		C/T	35 (34%)	106 (32,9%)	0,96 (0,60-1,54)	
G/G	7 (6,9%)	17 (5,3%)	0,72 (0,29-1,81)		A/A	18 (17,5%)	65 (20,2%)	0,91 (0,47-1,77)		C/C	2 (1,9%)	8 (2,5%)	1,27 (0,26-6,13)	
**Dominant**														
C/C	67 (65,7%)	226 (70,4%)	1,00	0,37	T/T	29 (28,2%)	115 (35,7%)	1,00	0,15	T/T	66 (64,1%)	208 (64,6%)	1,00	0,92
C/G-G/G	35 (34,3%)	95 (29,6%)	0,80 (0,50-1,29)		A/T-A/A	74 (71,8%)	207 (64,3%)	0,71 (0,43-1,15)		C/T-C/C	37 (35,9%)	114 (35,4%)	0,98 (0,62-1,55)	
**Recessive**														
C/C-C/G	95 (93,1%)	304 (94,7%)	1,00	0,56	T/T-A/T	85 (82,5%)	257 (79,8%)	1,00	0,54	T/T-C/T	101 (98,1%)	314 (97,5%)	1,00	0,75
G/G	7 (6,9%)	17 (5,3%)	0,76 (0,31-1,89)		A/A	18 (17,5%)	65 (20,2%)	1,19 (0,67-2,13)		C/C	2 (1,9%)	8 (2,5%)	1,29 (0,27-6,16)	
**Overdominant**														
C/C-G/G	74 (72,5%)	243 (75,7%)	1,00	0,53	T/T-A/A	47 (45,6%)	180 (55,9%)	1,00	0,07	T/T-C/C	68 (66%)	216 (67,1%)	1,00	0,84
C/G	28 (27,4%)	78 (24,3%)	0,85 (0,51-1,40)		A/T	56 (54,4%)	142 (44,1%)	0,66 (0,42-1,03)		C/T	35 (34%)	106 (32,9%)	0,95 (0,60-1,52)	

The best models for particular genes were as follows: for PPAR-y - the dominant model; for FTO – the overdominant model; for MC4R – the recessive model

Analysis of elevated blood pressure demonstrated statistically significant relationships in the codominant, dominant, and recessive models for the *PPAR-γ* polymorphism. In the codominant model, the C/C genotype inclined to increased blood pressure more than the C/G and G/G genotypes. In the dominant model, the C/C genotype predisposed to elevated blood pressure more than the C/G-G/G genotypes. In the recessive model, we observed a statistically significant relationship between the G/G genotype and a lower risk of increased blood pressure (Table 5).

**Table 5:** Odds ratios (OR) calculated assuming different models of inheritance of *PPARγ* *rs1801282*, *FTO rs9939609* and *MC4R* *rs17782313* SNPs with regard to blood pressure (S_5_^-^ = systolic blood pressure <130mmHg and/or diastolic blood pressure <85mmHg; S_5_^+^ **=** systolic blood pressure ≥130 mmHg or diastolic blood pressure ≥85 mmHg or related pharmacotherapy).

Genotype ***PPARy***		**S_5_^-^**	**S_5_^+^**	**OR**	**p**	Genotype ***FTO***	**S_5_^-^**	**S_5_^+^**	**OR**	**p**	Genotype ***MC4R***	**S_5_^-^**	**S_5_^+^**	**OR**	**p**
**Co-dominant**															
C/C	98 (63,2%)		195 (72,8%)	1,00	**<0,05**	T/T-A/T	56 (35,7%)	88 (32,8%)	1,00	0,84	T/T	103 (65,6%)	171 (63,8%)	1,00	0,89
C/G	43 (27,7%)		63 (23,5%)	0,74 (0,47-1,16)		A/T	71 (45,2%)	127 (47,4%)	1,14 (0,73-1,77)		C/T	50 (31,9%)	91 (34%)	1,10 (0,72-1,67)	
G/G	14 (9%)		10 (3,7%)	0,36 (0,15-0,84)		A/A	30 (19,1%)	53 (19,8%)	1,12 (0,64-1,97)		C/C	4 (2,5%)	6 (2,2%)	0,90 (0,25-3,28)	
**Dominant**															
C/C		98 (63,2%)	195 (72,8%)	1,00	**<0,05**	T/T	56 (35,7%)	88 (32,8%)	1,00	0,55	T/T	103 (65,6%)	171 (63,8%)	1,00	0,71
C/G-G/G		57 (36,8%)	73 (27,2%)	0,64 (0,42-0,98)		A/T-A/A	101 (64,3%)	180 (67,2%)	1,13 (0,75-1,72)		C/T-C/C	54 (34,4%)	97 (36,2%)	1,08 (0,72-1,64)	
**Recessive**															
C/C-C/G		141 (91%)	258 (96,3%)	1,00	**<0,05**	T/T-A/T	127 (80,9%)	215 (80,2%)	1,00	0,87	T/T-C/T	153 (97,5%)	262 (97,8%)	1,00	0,84
G/G		14 (9%)	10 (3,7%)	0,39 (0,17-0,90)		A/A	30 (19,1%)	53 (19,8%)	1,04 (0,63-1,72)		C/C	4 (2,5%)	6 (2,2%)	0,88 (0,24-3,15)	
**Overdominant**															
C/C-G/G		112 (72,3%)	205 (76,5%)	1,00	0,34	T/T-A/A	86 (54,8%)	141 (52,6%)	1,00	0,67	T/T-C/C	107 (68,2%)	177 (66%)	1,00	0,66
C/G		43 (27,7%)	63 (23,5%)	0,80 (0,51-1,26)		A/T	71 (45,2%)	127 (47,4%)	1,09 (0,73-1,62)		C/T	50 (31,9%)	91 (34%)	1,10 (0,72-1,68)	

The best models for particular genes were as follows: for PPAR-y - the recessive model; for FTO – the dominant model; for MC4R – the dominant and overdominant model

## DISCUSSION

The *PPAR-y* expression is noticeable in both endothelial and vascular smooth muscle cells, which has effects on its potential role in the regulation of vascular tension and blood pressure [9]. Essential information concerning the role of the *PPAR-y* mutation in the regulation of blood pressure has been provided by both human and animal studies.

Ostgren et al. maintain that the *PPAR-y* C/G mutation entails lower diastolic blood pressure in people. This relationship is probably independent of the influence that this mutation exerts on the functioning of the body [22]. Results of numerous studies show that the *PPAR-y* agonists (including thiazolidinediones) contribute to the lowering of blood pressure in animal models, which was also confirmed in patients with diabetes. It was established that hypotensive effect is at least partially independent of insulin-sensitizing activity [23,24]. Nonetheless, a decrease in blood pressure was considerably more apparent in animal models than in people [25]. In the study presented here, we noticed a tendency close to the results obtained by Ostgren et al. [22]. Both in the codominant and in the dominant model, the C/C genotype carriers were more inclined to have increased blood pressure than those with the C/G and G/G genotypes. In the recessive model, there was a relationship between the G/G genotype and a lower risk of elevated blood pressure in the group of perimenopausal women.

Review of research findings concerning the *PPAR-y* influence on the incidence of particular components of MetS shows that some authors regard the presence of the G variant as a protective factor against obesity [26]. Other reports, however, do not support this theory, showing that carriers of the C/G genotype are predisposed to increased BMI scores and obesity [27]. Swarbrick et al., on the other hand, did not find any connection between the C/G genotype and obesity, hypertension, or diabetes. Nevertheless, they made an interesting observation suggesting that abnormalities in the functioning of lipid metabolism were considerably more common among obese carriers of the G allele [28].

This result was partially confirmed in our study. We observed in the codominant model that the *PPAR-y* C/G genotype predisposed to hypertriglyceridemia slightly more than the C/C and G/G genotypes, which was also true in the overdominant model.

Over the recent decades, great interest in the impact of the *FTO* rs9939609 polymorphism on the occurrence of body weight related abnormalities [12,14–17], as well as lipid and carbohydrate metabolism [29] has been observed. The Kazakh cohort study demonstrated that polymorphisms in the *FTO* gene strongly predisposed to MetS. The carriers of the A allele were more likely to develop MetS-related abnormalities than their counterparts with the T allele [29]. These findings correspond with the results obtained by Freathy et al. [30].

Sikhayeva et al. reported on the relationship between type 2 diabetes and the *FTO* rs9939609 polymorphism in their cohort study of the Kazakh population. The authors observed that in the group with type 2 diabetes, the A allele was substantially more common than the T allele [29]. Muller et al., on the other hand, did not find any association between this gene and the parameters of the carbohydrate and lipid metabolism [31]. We established that in the recessive inheritance model, the *FTO* A/A genotype involved a higher risk of fasting hyperglycemia than the T/T-A/T genotypes.

The study of the multiethnic population comprised of Aboriginal, Chinese, European and South Asian participants living in Canada demonstrated that the presence of the A allele of the *FTO* gene entailed greater amount of fat mass and larger volume of subcutaneous adipose tissue [18]. Similar conclusions were drawn by the authors of the Slovak study, who established that the A/A genotype carriers had waist size higher by approximately 7.1 cm than the T/T genotype carriers [32]. This relationship was also observed (though only among white American women) by Song et al., who investigated postmenopausal women from various ethnic groups [33]. In our study, the relationships between particular inheritance models and waist size classified according to the IDF modified diagnostic MetS criteria from 2009 were not confirmed.

Available findings concerning the *FTO* impact on lipid metabolism are not unambiguous. Sikhayeva et al. found no confirmation for the relationship between the *FTO* polymorphism and the TG level, but reported on its association with the LDL level. The A/A genotype entailed significantly higher LDL levels compared to the A/T and T/T genotypes [29]. In our study, the A/T genotype in the overdominant model helped maintain an adequate HDL level, while the T/T-A/A genotypes were more often accompanied by HDL levels <50 mg/dl, but this relationship was not statistically significant.

Yang et al. claim that the *MC4R* polymorphisms affect the serum TG level and total cholesterol level in Chinese women at the mean age of 64.5 years. In their study, the C/C genotype carriers had significantly lower HDL levels than their counterparts with the T/T and T/C genotypes. The women with the T/T genotype had considerably lower TG levels than those with the C/C genotype. Still, the influence of particular *MC4R* polymorphisms on blood pressure was not demonstrated. In Yang’s study, a stable relationship between the C/C genotype and higher serum TG levels was obvious, even after exclusion of disturbing factors, such as age, BMI, and smoking [34]. The research conducted among the Japanese revealed that the minor C allele of the *MC4R* rs17782313 polymorphism had significant positive effects on the TG level, but was not related to obesity or BMI [35]. The study based on meta-analysis of the European population, on the other hand, provided evidence for the association between the *MC4R* polymorphisms and the risk of obesity [36]. The Genetic Investigation of ANthropometric Traits (GIANT) demonstrated that BMI scores were influenced by the C allele [37]. This result was not confirmed in the study of American Indians [38]. The above described relationships were not substantiated by our findings. We found no evidence for the contribution of particular genotypes to the development of MetS-related abnormalities.

Summing up, due to an increasing risk of serious functioning disorders and world growing incidence rate, MetS ― perceived as a set of abnormalities ― constitutes a serious public health problem. Apart from the lifestyle and environmental factors, MetS can develop as a result of genetic predisposition, which can be related to the polymorphic forms of genes that play an important part in the expression of MetS components, namely: *PPAR-γ*, *FTO* and *MC4R*. Our study provided evidence for the relationships between the A/A genotype of the *FTO* rs9939609 polymorphism and hyperglycemia, and between the C/C genotype of the *PPAR-γ* rs1801282 polymorphism and higher blood pressure.

### Limitations

The study sample was recruited through the distribution of information about the possibility of taking part in the study in the local environment, by means of recruitment was performed based on advertisement in local papers and information posters in public places. Despite our efforts, there is a risk that the study sample was not representative, which could affect the results.

## CONCLUSIONS

1. MetS-related abnormalities can be genetically determined, however the categorical division of symptoms according to the IDF criteria from 2009, allows for demonstrating only some of these relationships.

2. The A/A genotype of the *FTO* rs9939609 polymorphism increases the risk of hyperglycemia among women aged 45-60 years, and the C/C genotype of the *PPAR-γ* rs1801282 polymorphism involves higher blood pressure in this group.

## METHODS

The study sample consisted of 425 women. The criteria for inclusion in the study were: female sex, age between 45-60 years, the lack of current cancerous, psychiatric, or inflammatory diseases, and deliberate written consent to take part in the study. Those who failed to meet these criteria were excluded.

Recruitment was performed based on advertisement in local papers and information posters in public places. All participants came from the Westpomeranian Province (Poland). The majority of them (66.12%) lived in a city with the population of over 100,000, 11.29% lived in rural area, 6.12% lived in a city with the population of up to 10,000, and 16.47% lived in a city with the population of up to 100,000. 74.35% of the participants were professionally active, the rest of them were unemployed. 71.29% were married, 11.06% lived in cohabitation, and 17.65% were single. The majority of the women had second-level (44.94%) and had third-level education (39.53%). 2.59% had primary education, and 12.94% had vocational education. The mean age was 54.3 ± 4.2 years.

All subjects gave their informed consent for inclusion in the study. The study was conducted in accordance with the Declaration of Helsinki, and the protocol was approved by the Bioethical Commission of the [covered for blind review] (permission numbers KB-0012/181/13 and KB-0012/104/11).

### Description of the research procedure

The research procedure was divided into three stages. The first of them involved taking a history about basic sociodemographic data (age, place of residence, professional activity, education, marital status), and information concerning pharmacotherapy for hyperglycemia, hypertriglyceridemia, the HDL level, and blood pressure. The patients were also asked about their current cancerous, psychiatric, and inflammatory diseases.

At the next stage, anthropometric measurements were taken. Waist was measured in a standing position between the lower rib margin and the upper margin of the iliac crest at the end of a gentle exhalation. Blood pressure was gauged in a sitting position using a manual manometer. The cuff of the manometer was wrapped snugly around the patient's right upper arm at the heart level. The cuff was selected for the arm circumference.

Next, 6 ml ulnar venous blood samples were taken from each patient using the Vacutainer system. The blood was collected by qualified nurses in accordance with the relevant rules and procedures concerning collecting, storing, and transporting biological material. The levels of fasting glycemia, TG, and HDL were determined.

From the rest of the blood, DNA was isolated for genetic analysis of three gene polymorphisms: *PPAR-γ* rs1801283, *FTO* rs9939609, and *MC4R* rs17782313.

In accordance with the IDF diagnostics criteria from 2009, the results were categorized as positive (S^+^) if they deviated from the normal ranges thus reflecting the presence of abnormalities, and negative (S^-^) if they were within normal ranges (Table 6).

**Table 6:** The IDF modified diagnostic criteria for MetS from 2009, divided into negative (S-) and positive symptoms (S+).

**Symptom code**	**MetS criterion**	**Negative symptom S^-^**	**Positive symptom S^+^**
S_1_	Waist size	< 80 cm	≥ 80 cm
S_2_	Fasting glycemia	< 100 mg/dl (5.6 mmol/l)	≥ 100 mg/dl (5.6 mmol/l) or related pharmacotherapy
S_3_	TG level	< 150 mg/dl (1.7 mmol/l)	≥ 150 mg/dl (1.7 mmol/l) or related pharmacotherapy
S_4_	HDL cholesterol level	> 50 mg/dl (1.3 mmol/l)	≤ 50 mg/dl (1.3 mmol/l) or related pharmacotherapy
S_5_	Blood pressure	Systolic blood pressure < 130 and diastolic blood pressure < 85 mmHg	Systolic blood pressure ≥130 and/or diastolic blood pressure ≥ 85 mmHg or related pharmacotherapy

### Genotyping of the PPAR-γ rs1801282 (C>G), the FTO rs9939609 (T>A), and the MC4R rs17782313 (T>C) gene polymorphisms

Genomic DNA was isolated from whole blood in keeping with standard procedures. All genotyping was based on the real-time fluorescence resonance energy transfer performed using the Light Cycler System 1.0 (Roche Diagnostic, Poland). The gene polymorphisms were determined under the following conditions: polymerase chain reaction (PCR) was performed with 50 ng DNA in a total volume of 20 ml containing 2 ml reaction mix, 0.5 mM of each primer, 0.2 mM of each hybridization probe and 2 mM MgCl2 for 35 cycles of denaturation (95°C for 10min), annealing (60°C for 10 seconds), and extension (72°C for 15 seconds) as suggested by manufacturer. After amplification, a melting curve was generated by holding the reaction at 40°C for 20 seconds, and then heating slowly to 85°C. The fluorescence signal was plotted against temperature to give a melting curve for each sample.

The polymorphisms were determined based on the analysis of the melting curves. In the PPAR-γ *rs*1801282 (C>G) polymorphism, peaks were obtained at 53.14°C for the G allele and at 62.12°C for the C allele. In the *FTO rs*9939609 (T>A) polymorphism, peaks were obtained at 58.02°C for the A allele and at 63.08°C for the T allele. The fluorescence signal was plotted against temperature to give a melting curve for each sample. Peaks were obtained at 49.5°C for the T allele and at 58.23°C for the C allele.

### Statistical analysis

Statistical analysis was performed using STATISTICA 10.0 PL (StatSoft, Cracow, Poland,) and R environment. Statistical significance was set at a p value below 0.05. All tests were two-tailed. Nominal and ordinal data were expressed as percentages, whilst interval data were expressed as a mean value ± standard deviation. Nominal data were compared with X2 or Fisher exact test. In order to assess the relationship between MetS and genotypes four inheritance model were tested with Bayesian Information Criterion used to choose the best model (the smallest one). Odds ratio with confidence interval was used to show the influence of variables on MetS occurrence.
